# Causative agent distribution and antibiotic therapy assessment among adult patients with community acquired pneumonia in Chinese urban population

**DOI:** 10.1186/1471-2334-9-31

**Published:** 2009-03-18

**Authors:** Youning Liu, Minjun Chen, Tiemei Zhao, Hui Wang, Rui Wang, Baiqiang Cai, Bin Cao, Tieying Sun, Yunjian Hu, Qingyu Xiu, Xin Zhou, Xing Ding, Lan Yang, Jiansheng Zhuo, Yingchun Tang, Kouxing Zhang, Derong Liang, Xiaoju Lv, Shengqi Li, Yong Liu, Yunsong Yu, Zeqing Wei, Kejing Ying, Feng Zhao, Ping Chen, Xiaona Hou

**Affiliations:** 1 Department of Respiratory Diseases, Chinese PLA General Hospital, Beijing, PR China; 2 Department of Clinical Laboratory, Beijing Union Medical College Hospital, Beijing, PR China; 3 Clinical Pharmacological Laboratory, Chinese PLA General Hospital, Beijing, PR China; 4 Department of Respiratory Diseases, Beijing Union Medical College Hospital, Beijing, PR China; 5 Department of Respiratory Diseases, Beijing Hospital, Beijing, PR China; 6 Department of Clinical Laboratory, Beijing Hospital, Beijing, PR China; 7 Department of Respiratory Diseases, Changzheng Hospital, Second Military Medical College, Shanghai, PR China; 8 Department of Respiratory Diseases, First People's Hospital of Shanghai, Shanghai, PR China; 9 Department of Respiratory Diseases, First Affiliated Hospital of Xi'an Jiaotong University, Xi'an, PR China; 10 Department of Respiratory Diseases, Third Affiliated Hospital of Sun Yat-Sen University, Guangzhou, PR China; 11 Clinical Pharmacological Institutes, West China Hospital of Sichuan University, Sichuan, PR China; 12 Department of Respiratory Diseases, Second Affiliated University of China Medical University, Shenyang, PR China; 13 Department of Clinical Laboratory, Second Affiliated University of China Medical University, Shenyang, PR China; 14 Department of Infectious Diseases, First Affiliated Hospital of Zhejiang University, Zhejiang, PR China; 15 Department of Respiratory Diseases, Sir Run Run Shaw Hospital, Zhejiang, PR China; 16 Department of Respiratory Diseases, General Hospital of Shenyang Military Region, Shenyang, PR China; 17 Department of Clinical Laboratory, General Hospital of Shenyang Military Region, Shenyang, PR China

## Abstract

**Background:**

Knowledge of predominant microbial patterns in community-acquired pneumonia (CAP) constitutes the basis for initial decisions about empirical antimicrobial treatment, so a prospective study was performed during 2003–2004 among CAP of adult Chinese urban populations.

**Methods:**

Qualified patients were enrolled and screened for bacterial, atypical, and viral pathogens by sputum and/or blood culturing, and by antibody seroconversion test. Antibiotic treatment and patient outcome were also assessed.

**Results:**

Non-viral pathogens were found in 324/610 (53.1%) patients among whom *M. pneumoniae *was the most prevalent (126/610, 20.7%). Atypical pathogens were identified in 62/195 (31.8%) patients carrying bacterial pathogens. Respiratory viruses were identified in 35 (19%) of 184 randomly selected patients with adenovirus being the most common (16/184, 8.7%). The nonsusceptibility of *S. pneumoniae *to penicillin and azithromycin was 22.2% (Resistance (R): 3.2%, Intermediate (I): 19.0%) and 79.4% (R: 79.4%, I: 0%), respectively. Of patients (312) from whom causative pathogens were identified and antibiotic treatments were recorded, clinical cure rate with β-lactam antibiotics alone and with combination of a β-lactam plus a macrolide or with fluoroquinolones was 63.7% (79/124) and 67%(126/188), respectively. For patients having mixed *M. pneumoniae *and/or *C. pneumoniae *infections, a better cure rate was observed with regimens that are active against atypical pathogens (e.g. a β-lactam plus a macrolide, or a fluoroquinolone) than with β-lactam alone (75.8% vs. 42.9%, *p *= 0.045).

**Conclusion:**

In Chinese adult CAP patients, *M. pneumoniae *was the most prevalent with mixed infections containing atypical pathogens being frequently observed. With *S. pneumoniae*, the prevalence of macrolide resistance was high and penicillin resistance low compared with data reported in other regions.

## Background

Community-acquired pneumonia (CAP) remains a common disease with high mobidity, mortality, and treatment cost [1,2]. The microbial patterns reported for CAP differ considerably, depending on epidemiologic area, patient populations, and the extent and nature of the microbiologic techniques used. Knowledge of predominant microbial patterns in CAP constitutes the basis for initial decisions about empirical antimicrobial treatment [3]. However, no prospective study has been done to investigate such patterns in a large Chinese population over several districts at the same time.

In the present work, we conducted a prospective study of the etiology of CAP in adult patients at 12 centers in 7 Chinese cities to assess the causative microbial spectrum, the influence of patient age, previous antibiotic use, and the Pneumonia Patient Outcomes Research Team (PORT) category of pneumonia on microbial patterns in the disease [4]. Antimicrobial susceptibility of isolates from clinical specimens was also tested to estimate the prevalence of drug resistance of common CAP-causing bacteria, especially *Streptococcus pneumoniae *and *Haemophilus influenzae*. Meanwhile the outcome of antibiotic therapy for CAP was analyzed. The results revealed a different predominant microbial spectrum and relatively higher penicillin susceptibility of *S. pneumoniae *in the Chinese population than previously reported [5,6].

## Methods

### General information

A prospective study of the causes and antimicrobial treatment outcome of CAP was conducted in consecutively enrolled adult patients (age ≥ 18 years) between 1 December 2003 and 30 November 2004 at 12 centers in 7 Chinese cities (Beijing, Shanghai, Shenyang, Xi'an, Chengdu, Guangzhou, and Hangzhou). The cities were selected according to their geographical locations: Beijing and Shenyang lie in the north area, Guangzhou in the south area, Shanghai and Hangzhou in the east area, and Xi'an and Chengdu in the west area. The study year was divided into four periods: December to February, March to May, June to August, and September to November. Two central laboratories, PLA General Hospital laboratory and Beijing Union Medical College Hospital laboratory, conducted pathogen identification and drug susceptibility testing. Therapy was recommended according to the CAP guidelines from the Chinese Medical Association [7], but the final drug choice was determined by attending physicians. Informed consent was obtained from each patient before sputum and blood sample collection. The protocol was approved by the Ethics Committee for Clinical Studies at PLA General Hospital (Protocol: 2003-#035).

### Diagnostic criteria

According to the guidelines from the Chinese Medical Association [7], CAP was defined as an acute infiltrate on a chest radiograph and at least one of the following: (a) new or escalating cough, sputum production, purulent sputum, chest pain; (b) fever; (c) altered breath sounds or/and rales; (d) white blood cell count: > 10 × 10^9^/L or < 4 × 10^9^/L. Patients were excluded if they had tuberculosis, lung cancer, non-infective interstitial lung diseases, pulmonary oedema, pulmonary lobe collapse, pulmonary thromboembolism, pulmonary infiltration with eosinophilia, or pulmonary angiitis. Atypical pathogens defined as *Mycoplasma pneumoniae*, *Chlamydophila pneumoniae*, and *Legionella spp*. The microbial etiology of pneumonia was considered if one of the following criteria was met: (1) valid sputum sample generating one or more predominant microbial pathogens; (2) blood culture producing a bacterial pathogen; (3) serologic conversion: e.g. a ≥ 4-fold increase in titers of antibodies to *Mycoplasma pneumoniae*, *Chlamydophila pneumoniae*, *Legionella pneumophila*, or respiratory viruses (influenza virus A and B, adenovirus, and respiratory syncytial virus).

### Microbiological evaluation

Regular sputum sampling was done with most (590/610) patients enrolled in the study, and blood samples were taken if patients had a fever of > 38.5°C. Sputum was Gram-stained. Representative sputum originated from the lower respiratory tract was defined as that containing > 25 granulocytes and < 10 epithelial cells per low power field microscopic view. Validated sputum and blood samples were cultured; microorganisms were identified according to standard methods [8].

Blood samples (6–8 ml) were collected aseptically from patients. Acute sera were separated and stored at -80°C until convalescent sera, obtained 2 to 4 weeks after the acute specimen, were available for simultaneous testing [9]. The antibody titer to *M. pneumoniae *was determined by microparticle agglutination using the Serodia Myco^® ^commercial kit (Fujirebio, Japan). *C. pneumoniae*-specific IgG antibody titers were determined by the indirect immunofluorescence method using the FI2192-1010G commercial kit (Euroimmun, Germany), as were antibodies to 14 different serogroups of *L. pneumophila*. Respiratory virus antibodies (IgG) were detected by ELISA using the Virion-Serion commercial kit (Febricant, Germany).

### Antimicrobial susceptibility testing

MICs of isolates were determined at Beijing Union Medical College Hospital laboratory, using agar dilution according to CLSI protocols. Breakpoint concentrations used to interpret MIC data qualitatively were based upon those published by the National Committee for Clinical Laboratory Standards of the USA (NCCLS, 2004) [10]. The inocula comprised 10^4^–10^5 ^cfu/spot, delivered with a multipoint inoculator. Cultures were incubated with antimicrobial agents selected on the basis of common empiric therapy options in China.

### Data collection

Clinical, radiographic, and laboratory data were recorded on a data sheet and entered into a computer database. The following data were recorded on enrolling: age, gender, comorbidities, antimicrobial treatment prior to enrolment, duration of symptoms before the diagnosis of pneumonia, clinical symptoms (body temperature, pleuritic chest pain, purulent sputum), vital signs (respiratory rate, heart rate, arterial systolic and diastolic blood pressure, presence of rales), haematology (total WBC with differential counts, platelet count, hemoglobin), chest radiographic pattern, and alcohol consumption.

Data on modifying or risk factors collected, included underlying cardiopulmonary diseases, exposure to a child in a day care center, residence in a nursing home, β-lactam therapy within the past 3 months, broad-spectrum antibiotic therapy for > 7 d in the past two months, corticosteroid therapy (> 10 mg of prednisone per day), alcoholism, previous pneumonia history, and malnutrition.

### Antibiotic therapy analysis

According to the causative pathogens identified, we classified the patients into four groups for analysis of outcome of antibiotic therapy. First group: *M. pneumoniae *and *C. pneumoniae *mono- and co-infection; second group: bacterial co-infection with *M. pneumoniae*, *C. pneumoniae *or both; third group: *L. pneumophila *mono-infection or mixed with other pathogens; and fourth group: bacterial infection only. Clinical response, evaluated post-treatment, was rated as cure (complete resolution of all signs and symptoms of pneumonia), improvement, or lack of progression for all abnormalities on chest radiograph [11].

### Statistical analysis

Statistical analysis was carried out using SAS (SAS Institute, Cary, NC, USA) version 8.2. Results are presented descriptively (some expressed as mean ± SD). Data were compared by use of the χ^2 ^statistic or Fisher's exact test. For all analyses, *p *< 0.05 was considered to be significant.

## Results

### General information

The 610 enrolled patients (382 male and 228 female) had a mean age of 52.4 (± 20.0 SD) years. When stratified into the five classes of the PORT cumulative point system [4], most patients were in low-risk Classes I (33.8%), II (36.2%) and III (15.9%); moderate-risk Class IV disease was determined in 13.1% of patients, and high-risk Class V in only 1.0%. Clinical symptoms, as well as modifying and risk factors, are summarized in Table 1. Three hundred forty two patients (56.1%) received antimicrobial treatment prior to enrollment. Thirty eight percent of patients had at least one co-morbid illness, with cardiac co-morbidity being the most frequent condition (25.9%), followed by pulmonary co-morbidity (12.8%).

**Table 1:** Summary of characteristics of all enrolled patients with community-acquired pneumonia (n = 610)

	n (%)*
**Symptoms**	
Fever	455 (74.6)
Cough	582 (95.4)
Purulent sputum	536 (87.9)
Rales	402 (65.9)
Leukocytosis > 10 × 10^9^/L	260 (42.6)
**Modifying factors**	
Exposure to a child in a day care center	7 (1.2)
β-lactam therapy within the past 3 months	120 (19.7)
Broad-spectrum antibiotic therapy for > 7 d in the past two months	61 (10.0)
Corticosteroid therapy (> 10 mg of prednisone per day)	9 (1.5)
Alcoholism	1 (0.2)
History of pneumonia	79 (13.0)
**Comorbidities**	
Cardiac	158 (25.9)
Pulmonary	78 (12.8)
Diabetes mellitus	33 (5.4)
Central nervous system	25 (4.1)
Renal	8 (1.3)
Hepatic	7 (1.1)

*Patient number, numbers in parenthesis are percentage of total patient number.

### Pathogens distribution

Of 610 patients, sputum samples were obtained in 590 patients, and blood samples for culture were available in 141 patients. Of the 590 patients with sputum samples, *S. pneumoniae *was the most common bacterium identified, accounting for 10.3%, followed by *H. influenzae *(9.2%).

Overall, non-viral pathogens were detected in 324 of the 610 patients (Table 2). Of these, 195 were detected by sputum examination, 5 by hemoculture, and 191 by serology testing. The most common agent identified was *M. pneumoniae*, which was found in 126 patients (20.7%). *S. pneumoniae *was identified in 63 patients (10.3%), followed by *H. influenzae *in 56 patients (9.2%). The frequency of other infective agents was 6.6% for *C. pneumoniae *(40 patients), 6.1% for *Klebsiella pneumoniae *(37 patients), 5.1% for *L. pneumophila *(31 patients), 3.8% for *Staphylococcus aureus *(23 patients), 1.6% for *Escherichia coli *(10 patients), 1.3% for *Moraxella catarrhalis *(8 patients), and 1.0% for *Pseudomonas aeruginosa *(6 patients). Atypical pathogens accounted for 31.3% overall. In 184 patients whose sera antibody response to respiratory viruses were analyzed, 35 patients (19.0%) were tested positive for at least one viral infection. The distribution of viruses was listed in Table 3. Overall, adenovirus was the most common, identified in 16 patients (8.7%), followed by influenza virus B in 12 patients (6.5%), respiratory syncytial virus in 8 patients (4.3%), and influenza virus A in 5 patients (2.7%). In patients that tested positive for viral infections, mixed infections with an atypical pathogen or a bacterium were detected in 23 patients (65.7%), accounting for 12.5% of the total 184 patients tested for viral infection. The most common pathogen involved in co-infection with virus was *M. pneumonia*, accounting for 8 patients. The most common bacterium found in co-infection was *H. influenzae*, accounting for 4 patients.

**Table 2:** Non-viral pathogens detected among 610 patients with community-acquired pneumonia

Pathogen	Patients, n (%)
**Mono-infection**	
*Mycoplasma pneumoniae*	82 (13.4)
*Streptococcus pneumoniae*	37 (6.07)
*Haemophilus influenzae*	33 (5.4)
*Chlamydophila pneumoniae*	29 (4.8)
*Klebsiella pneumoniae*	23 (3.8)
*Staphylococcus aureus*	17 (2.8)
*Legionella pneumophila*	17 (2.8)
*Escherichia coli*	6 (1.0)
*Pseudomonas aeruginosa*	5 (0.8)
*Moraxella catarrhalis*	5 (0.8)
**Mixed infections**	70 (11.5)
Two pathogens	64 (10.5)
Three pathogens	5 (0.8)
Four pathogens:	1 (0.2)
**Total number of patients with identified pathogens**	324 (53.1)

Note: Percentage of the most three common agents in which pathogens were identified was 38.9% for *M. pneumoniae*, 19.4% for *S. pneumoniae*, and 17.3% for *H. influenzae*.

**Table 3:** Respiratory viruses detected among 184 patients with community-acquired pneumonia

Pathogen	Patients, n (%)
**Mono-virus infection**	
influenza virus B	6 (3.3)
adenovirus	3 (1.6)
respiratory syncytial virus	2 (1.1)
influenza virus+ adenovirus	1 (0.5)
**Mixed infections**	
**Two pathogens**	15 (8.2)
**Three pathogens**	6 (3.3)
**Four pathogens**	2(1.1)
**Total**	35 (19.0)

Note: Percentage of adenovirus in which viruses were identified was 45.7%, and 34.3% for influenza virus B, 22.9% for respiratory syncytial virus, 14.3% for influenza virus A.

In addition to co-infection with respiratory viruses, mixed infections were present in 70 of the 610 patients whose microbial etiology was determined. Among these 70 patients, co-infection with two, three, and four pathogens was detected in 64 (91.4%), 5 (7.1%), and 1 (1.4%) patients, respectively. Among the 63 patients from whom *S. pneumoniae *was identified, co-infection was detected in 26 (41.3%) patients (i.e.: *M. pneumoniae *in 15 (23.8%), *C. pneumoniae *in 3 (4.8%), and other pathogens in 8 (12.7%)). Where *M. pneumoniae *infection was detected, a co-pathogen was diagnosed in 44 (34.9%) cases (*S. pneumoniae *in 15, *H. influenzae *in 13, and other pathogens in 16). Among the 195 patients with bacterial infection, an atypical co-pathogen was identified in 62 (31.8%) cases.

*M. pneumoniae *were found less often in the elderly than in patients aged ≤ 50 years (13.4% vs. 30.0%, *p *< 0.05). Co-morbidity was found in 27.8% of *M. pneumoniae*-infected patients. Age or co-morbidity alone had no impact on *C. pneumoniae *infection, but *C. pneumoniae *was more frequently found in elderly patients (> 70 years) without co-morbid illness than in those with co-morbidity (18.4% (7/38) vs. 6.3% (7/112), *p *< 0.05). Respiratory virus infection was less frequent in patients aged > 70 years than in patients ≤ 70 years old (0% vs. 24.6%, *p *< 0.05). In patients aged > 50 years, *K. pneumoniae *infection was more frequent than in those aged ≤ 50 years (8.5% vs. 3.0%, *p *< 0.05). Five of the 6 patients with *P. aeruginosa *infection were aged > 70 years. In patients with cardiovascular or pulmonary co-morbidity, 12.4% (21/170) had *K. pneumoniae *or *P. aeruginosa *infections, compared to only 5.0% (22/440) of patients without these co-morbidities (*p *< 0.05).

56.1% of patients received antibiotics before enrolling. *H. influenzae *was 6-fold more frequently isolated from patients who had not received antibiotics before enrolling than those who had been treated with antibiotics (17.2% (46/268) vs. 2.9% (10/342), *p *< 0.05). Prior antibiotic therapy did not significantly reduce the frequency of *S. pneumoniae *isolation.

Patients of PORT Classes I and II were more likely to have *S. pneumoniae *and *H. influenzae *etiology. In patients with Class V disease, association with *K. pneumoniae *was evident. *M. pneumoniae *was common in Class I, II, and III disease, especially in Class I. With regard to seasonal variations, isolation rate of common pathogens did not show any statistically significant differences in the four period of our survey.

### Antimicrobial susceptibility

A total of 63 isolates of *S. pneumoniae *were submitted for antimicrobial susceptibility testing. The proportions of intermediately or fully resistant isolates to penicillin were 19.0% and 3.2%, respectively. The prevalence of isolates considered non-susceptible to azithromycin and new fluroquinolones were 79.4% and ≤ 6.3%, respectively (Table 4). In 56 isolates of *H. influenzae *tested, 8.4% produced beta-lactamase and 11.9% were non-susceptible to ampicillin.

**Table 4:** Antimicrobial susceptibility of 63 *S. pneumoniae *isolates obtained in the study

Antimicrobial agent	% of isolates			MIC (μg/ml)		
		
	Susceptible	Intermediate	Resistant	MIC_50_	MIC_90_	Range
Penicillin	77.8	19.0	3.2	0.016	0.5	0.016–4
Azithromycin	20.6	0.0	79.4	256	> 256	0.032–512
Amoxycillin/clavulanic acid	95.2	3.2	1.6	0.032	1	0.032–8
Levofloxacin	93.7	0.0	6.3	1	2	0.5–16
Gatifloxacin	93.7	0.0	6.3	0.25	0.5	0.125–4
Moxifloxacin	95.2	3.2	1.6	0.125	0.25	0.064–4

### Antibiotics usage and clinical outcome

In our study, the most frequently administered antibiotics were β-lactams, accounting for 73.6% (449/610), followed by fluoroquinolones (49.8%, 304/610) and macrolides (22.8%, 139/610). There was a total of 315 patients whose infecting pathogen was identified and antibiotic usage was recorded. Since only 3 patients used a macrolide as monotherapy, the clinical outcome of these patients was not considered. In the other 312 patients, the clinical cure rate with β-lactam antibiotics was 63.7% (79/124), with combination of a β-lactam plus a macrolide or with fluoroquinolone (either alone or in combination with other antibiotics) was 67% (126/188). For patients affected by atypical pathogens, cure rates are presented in Table 5. For mixed infections involving bacteria plus *M. pneumoniae *and/or *C. pneumoniae*, β-lactams plus a macrolide or fluoroquinolone (alone or combined with other antibiotics, e.g. regimens that are active against atypical pathogens) showed a statistically significant higher cure rate than β-lactams alone (75.8% v.s. 42.9%, *p *= 0.045). In patients with co-morbid illness, the total cure rate of therapy with the combination of a β-lactam plus a macrolide or with fluoroquinolone was not significantly greater than that with β-lactams (40.4% vs. 50.0%, *p *> 0.05).

**Table 5:** Cure rate [n (%)] of antibiotics in patients with CAP affected by atypical pathogens

Pathogens	β-lactam alone	β-lactam plus macrolides or fluoroquinolone (alone or combine with other antibiotics)
MP, CP, MP+CP	25(62.5)	45(73.8)

MP+bacteria, CP+bacteria, MP+CP+bacteria	6(42.9)	25(75.8*)

LP or LP mixed with other pathogens	8(61.5)	10(55.6)

MP: *Mycoplasma pneumoniae*; CP: *Chlamydophila pneumoniae*; LP: *Legionella pneumophila*

*compared to β-lactam alone, *p *= 0.045

## Discussion

The distribution of pathogens associated with CAP is attracting world-wide attention. The present study is the first in China to prospectively investigate the pathogen distribution in a large, broadly distributed patient population. The study comprised several representative districts and covered all seasons of the year. The data reveal a unique pattern of pathogen distribution in CAP patients in China and show a relatively higher susceptibility of *S. pneumoniae *isolates to penicillin but relatively lower susceptibility to fluoroquinolones than that reported in Europe and North America.

In previous studies, 30 to 60% of the CAP cases did not yield an identifiable pathogen [12], in line with our results. Although *S. pneumoniae *remains the most prevalent or frequently isolated etiologic agent in cases of CAP [2,13], other organisms, such as *H. influenzae *and *M. catarrhalis*, as well as the so-called atypical pathogens, including *M. pneumoniae*, *C. pneumoniae*, and *L. pneumophila*, are now being reported more frequently than in the past [14,15]. Atypical pathogens are responsible for 30 to 40% of cases of CAP [16]. In some reports, the infection with atypical pathogens was more than with *S. pneumoniae*, especially for *M. pneumoniae*. Moola et al [15] reported that when pathogens were confirmed in 131 of 504 patients, *M. pneumoniae *was found in 25% patients, and *S. pneumoniae *in 22%. In a study performed in Spain [16], *M. pneumoniae *was the most frequent pathogen (33%), with *S. pneumoniae *being found in only 19 of 110 patients with CAP.

In our study, the rate of atypical pathogen infection is 31.3% (if convalescent sera obtained longer than 4 weeks after the acute specimen, *L. pneumophila *may be more, and this percentage may be higher); *M. pneumoniae *is the most prevalent etiologic agent, accounting for 20.7% of CAP cases, i.e. more than those associated with *S. pneumoniae*. Such finding is similar to our previous study between 2001 and 2002 in Beijing [9,17]. Previous reports show that viral infection in patients with CAP varies from 4% to 39% [18], the most frequent being influenza spp. In the present study, respiratory viruses account for 19.0% of tested patients, with adenovirus being the most common.

Previous studies have shown that some patients with CAP can have mixed infections involving both bacterial and atypical pathogens [2,19]. *C. pneumoniae *is frequently found in mixed infections and has been detected in over half of cases with *S. pneumoniae *in reports from other countries [2,19]. In our study, however, *M. pneumoniae *is the most frequent co-infecting pathogen. Multiple simultaneous infections might interfere with the pulmonary cleansing function and thus help establish setting for CAP [20]. Pathogens such as *C. pneumoniae *induce ciliostasis in human bronchial epithelial cells [21], and *M. pneumoniae *exerts a toxic effect on ciliated human epithelium [22]. Currently, the clinical implications of mixed infections are still undetermined. Influenza and other viruses can cause primary viral pneumonia; secondary bacterial infections are common in hospitalized adults, and the reported frequency has ranged widely, from 26% to 77% in different studies [18]. The common cause of bacterial superinfection is *S. pneumoniae*. In our study, mixed infection of respiratory viruses with other pathogens accounts for 12.5%. The most common co-infecting pathogen was *M. pneumoniae*.

The impact of age is restricted to an association of younger patients (age ≤ 50 years) with *M. pneumoniae*. This finding is consistent with several previous studies [23,24], which show that *M. pneumoniae *is the most common etiologic agent in the 17–44 year age group. Patients without co-morbid illnesses are also more likely to have *M. pneumoniae *infection. In our study, only 27.8% patients of *M. pneumoniae *have co-morbid illnesses. It has been reported that *C. pneumoniae *infection is more likely to occur in elderly persons with co-morbid disease than in those who are otherwise healthy [25]. However, our data showed a higher incidence of *C. pneumoniae *infection in older patients without co-morbid illness than with, contradicting previous conclusion. An association of older age with Gram-negative pathogens has been observed by several groups [12,26]. It is well known that colonization of the oropharyngeal mucosa by aerobic Gram-negative bacilli increases with age [27]. In our study an age of > 50 years was significantly associated with these pathogens. Pulmonary co-morbidity also predisposes to pneumonia due to *K. pneumoniae *or *P. aeruginosa*, probably due to previous tracheabronchial colonization in COPD [28]. In our study, *Staphylococcus aureus *was more frequent in PORT I than in PORTs II through IV, probably due to patients having low co-morbidities of diabetes mellitus and to sample size being too small.

Antibiotic resistance in pneumococci is now considered to be a global problem. The ANSORP (Asian network for surveillance of resistant pathogens) study, conducted in Asian countries from September 1996 to June 1997, showed that the non-suscepibility to penicillin was as high as 79.7% in Korea and non-susceptibility to erythromycin was as high as 90.5% in Taiwan [29]. In China, penicillin-non-susceptibile *S. pneumoniae *ranged from 13.9% to 42.7%, and erythromycin-non-susceptibile *S. pneumoniae *ranged from 42.5% to 83.6% in surveys conducted from 1997 to 2003 [5,6,14,30-32]. Among the 63 strains of *S. pneumoniae *obtained from the present study, non-susceptibility to penicillin was 22.2%; non-susceptibility to azithromycin was 79.4%. In 2008 year, CLSI changed the break point of penicillin for patients with non-CNS infections, according to this, the non-susceptibility to penicillin was only 3.2%. Although new fluoroquinolones have good activity against *S. pneumoniae*, the resistance rate of new fluoroquinolones is higher in China than that reported in other countries except for Hong Kong [33], which is possibly a result of selective pressure due to increased quinolone use in China.

American Thoracic Society guidelines [34] advocate that all populations with CAP should be treated for possible infection with atypical pathogens. Macrolides are recommended as the first-line choice for outpatients. However, such a recommendation may not be suitable for patients in China because the prevalence of macrolide resistance is so high (about 70% among *S. pneumoniae *isolates) and because *erm*B-mediated high-level resistance is more frequent than low-level resistance conferred by *mef*A (79.1% contained the *erm*B gene, 10.8% contained the *mef*A gene, and 10.1% harbored both the *erm*B and *mef*A genes) as shown in our previous study [6]. In the present study, for patients infected with *M. pneumoniae *and/or *C. pneumoniae*, the cure rate with β-lactams plus macrolides or with fluoroquinolones was not significantly better than with β-lactams alone. A meta-analysis also showed no advantage of antibiotics active against atypical pathogens over β-lactams [35]. Thus, β-lactams (penicillin, amoxicillin, cephalosporins) should remain agents of choice in the initial management of mild CAP without co-morbidity in China. But for moderate or severe infection or in patients with co-morbidities, therapy should be recommended with either a β-lactam/macrolide combination or an anti-pneumococcal fluoroquinolone alone [36-39].

## Conclusion

In Chinese adult CAP patients, *M. pneumoniae *was the most prevalent with mixed infections containing atypical pathogens being frequently observed. With *S. pneumoniae*, the prevalence of macrolide resistance was high and penicillin resistance low compared with data reported in other regions. β-lactams (penicillin, amoxicillin, cephalosporins) should remain agents of choice in the initial management of mild CAP without co-morbidity in China.

## Competing interests

The authors declare that they have no competing interests.

## Authors' contributions

YL, TZ, BC, BC, TS, QX, XZ, LY, YT, DL, SL, YY, KY, and PC conceived the study, enrolled patients and collected data. MC, HW, RW, YH, XD, JZ, KZ, XL, YL, ZW, FZ, and XH conducted microorganisms identification, and HW responsible for antimicrobial susceptibility testing. All authors read and approved the final manuscript.

## Pre-publication history

The pre-publication history for this paper can be accessed here:

http://www.biomedcentral.com/1471-2334/9/31/prepub
